# Long-term complications of sulfur mustard poisoning: retinal electrophysiological assessment in 40 severely intoxicated Iranian veterans

**DOI:** 10.1186/s40942-017-0059-x

**Published:** 2017-03-06

**Authors:** Nasser Shoeibi, Mir Naghi Mousavi, Mahdi Balali-Mood, Mohammad Moshiri, Emadodin Darchini-Maragheh, Seyed Reza Mousavi, Mojtaba Abrishami

**Affiliations:** 1 Retina Research Center, School of Medicine, Mashhad University of Medical Sciences, Mashhad, Iran; 2 Eye Research Center, School of Medicine, Mashhad University of Medical Sciences, Mashhad, Iran; 3 Medical Toxicology Research Center, School of Medicine, Mashhad University of Medical Sciences, Mashhad, Iran; 4 Department of Clinical Toxicology, Imam Reza Hospital, School of Medicine, Mashhad University of Medical Sciences, Mashhad, Iran; 5 Department of Pharmacodynamy and Toxicology, School of Pharmacy, Mashhad University of Medical Sciences, Mashhad, Iran; 6 Student Research Assembly, Mashhad University of Medical Sciences, Mashhad, Iran; 7 Eye Research Center, Farabi Eye Hospital, Tehran University of Medical Sciences, Qazvin Square, Tehran, 1336616351 Iran

**Keywords:** Sulfur mustard, Electroretinograghy, Electrooculography, Chemical warfare agents, Retina

## Abstract

**Background:**

The eye is one of the most sensitive organs to sulfur mustard (SM) [C_4_H_8_Cl_2_S], and preliminary symptoms of exposure usually become evident in the eyes. In this study we aim to evaluate the possible long-term retinal electrophysiologic complications of SM poisoning in Iranian veterans during Iran–Iraq war (1980–1988).

**Methods:**

In a cross-sectional study forty Iranian veterans who were exposed to mustard gas during the Iran–Iraq war (1980–1988) were included. All the cases underwent complete ocular exam and retinal electrophysiological evaluation, including electroretinography (ERG) and electrooculography (EOG). Data was analyzed using SPSS software. The normal distribution was checked using the Shapiro–Wilk test. Comparison of electrophysiologic values with maximum standard levels was performed using one-sample Student t-test and test of significance was one-tailed.

**Results:**

Foreign body sensation (70%), dry eye (50%), photophobia (30%), lacrimation (20%) and pain sensation (10%) were among the common symptoms. ERG showed significant reduced amplitude in rod response, maximal combined response, oscillatory potentials, cone response and 30 Hz flicker waves compared to normal values (*p* < 0.05). Implicit time of b-wave rod response ERG recording was significantly decreased (*p* < 0.05). Implicit time of cone response b-wave was within normal limits. In EOG, Arden ratio did not decrease (total average of 2.311 and 2.48 in right and left eyes, respectively).

**Conclusion:**

Delayed toxic effects of SM poisoning in the veterans were observed in the retina, but not in the retinal pigment epithelium layer. As the retina is a neural tissue, long-term effects of SM on neural tissues are presumed.

## Background

Sulfur mustard (SM) [C_4_H_8_Cl_2_S], is among the most potential alkylating chemical weapons and is known as one of the weapons of choice in modern tactical warfare. It has caused many casualties, especially in Iran–Iraq war during 1980–1988 [1]. SM exposure primarily affects ocular tissue, respiratory tract and skin [2, 3]. The eye is the most sensitive organ to SM and preliminary symptoms of exposure usually become evident in the eyes [4–6].

Acute ophthalmic symptoms usually begin with ocular pain, lacrimation and photophobia. Physical examination may reveal eyelid spasm, swelling and edema of the peri-orbital skin, conjunctival injection, and inflammation of the anterior chamber [7–10]. Intraocular pressure (IOP) may increase and remain elevated for a few days [11]. Superficial punctate keratitis, superficial infiltration, corneal abrasion, whorl pattern dystrophy and corneal ulcer have been reported as immediate corneal effects of SM in exposed patients [12]. Electroretinographic findings in an animal model showed no abnormality 6–7 weeks after exposure [11].

Long-term ocular complications of SM are also noticeable. Severe long-term ocular effects of SM on various organs have been reported in both the World War I survivors and in Iranian veterans, three decades after initial exposure [6, 13].

Although SM-related chronic ocular complications in anterior segment of eyes have been previously reported, retinal assessment, to our knowledge, has not been addressed yet. On the other hand, looking back to the new surge of terror activity in recent decades, deployment of SM attacks and ease of its production, raise the concern that it can be used again in the war and even by terrorists anywhere and anytime and will pose a threat to international security and peace.

In this study, we have sought to determine long-term ocular complications of SM poisoning in Iranian veterans with a special survey of retinal involvement, by clinical examinations and electrophysiological studies.

## Methods

### Study design and participants

It was a cross-sectional analytic study on 40 Iranian veterans who were exposed to mustard gas during the Iran–Iraq war (1980–1988). It is a pilot study and we aimed to evaluate posterior segment complications of mustard gas in a cooperative group of veterans. Informed consent was obtained from each participant after the nature of the experimental procedures had been explained. This study was carried out in accordance with ethical standards set forth by the 1989 Declaration of Helsinki with the approval of the Institutional Review Board and Ethics Committee of Mashhad University of Medical Sciences. It was carried out also in coordination with the Veterans and Martyrs Affair Foundation (VMAF). Eligible participants had a history of exposure to SM during the Iran–Iraq war and were diagnosed as severely intoxicated veterans with more than 25% of disabilities due to complications of SM poisoning. The disability percentages are determined regularly by VMAF according to severity of complications of SM in different organs. All the registered veterans were completely notified about the procedure, and signed the written informed consent, otherwise the participants with disinclination were excluded. In order to avoid the interference of lenticular nuclear sclerosis, only patients with clear lens or pseudophakic eyes were included.

Other exclusion criteria were: family history of heritable retinal diseases, systemic diseases affecting the retina, visual acuity of less than 20/200 in Snellen chart, refractive errors (spherical equivalent of more than 3 diopters), intraocular pressure and visual fields suggesting glaucoma.

The study took place at the Retina Research Center of Khatam Al-Anbia Eye Hospital and Medical Toxicology Research Center in Mashhad University of Medical Sciences, Mashhad, Iran from April 2011 to February 2012.

### Patients’ evaluations

Every week, two SM-exposed veterans were recruited from VMAF to Retina Research Center. Demographic data, chemical warfare agents (CWA) exposure information and clinical history as well as complete ophthalmic examination were carried out by an ophthalmologist, and data were recorded in a pre-designed form.

In the next step, all the patients underwent electrophysiological evaluations, including electroretinograghy (ERG) and electrooculography (EOG). Standardized full-field ERGs were elicited with Ganzfeld stimuli using the commercial ERG system (Retiport32; Roland Consult) according to International Society for Clinical Electrophysiology of Vision (ISCEV) guidelines (standard flash, 3.0 cd/s/m^2^). For the recordings, pupils of both eyes were maximally dilated with 0.5% tropicamide and 0.5% phenylephrine, and each eye was examined separately; while the other eye was occluded. A DTL-electrode was applied, and the ground electrode was attached to the forehead. The rod response ERG was recorded after 30 min of dark adaption. For the cone response ERG, the background luminance was set at 34 cd/m^2^. An adjustable voltage window was used to reject artifacts. Amplitudes and implicit times were measured according to the ISCEV recommendations.

EOG was performed according to Arden and Kelsey’s methods [14]. Eye movements were recorded using surface electrodes placed at the lateral and medial canthi of each eye. Thirty degree eye movements for 10 s each minute were recorded during 15 min of dark adaptation, which followed by 10–15 min in full-field (Ganzfeld) light adaptation (100 cd/m^2^). Dark trough and light peak amplitudes were measured, and Arden ratios were calculated. Ratios of 180% and more were considered normal.

ERG and EOG recordings were obtained from both eyes. Full-field ERG and EOG testing were performed in the morning for all the veterans by one experienced technician.

### Data analysis

Data was analyzed using SPSS (Statistical Package for Social Sciences, 11.5; Chicago, IL, USA) by a statistician who was blinded to participants’ identity. Data cleaning was done to check the quality of data set, probable outliers and missing data. Data set was summarized using frequency tables and boxplots for qualitative variables and also descriptive statistics and histograms for quantitative variables. The normal distribution of all variables was checked using the Shapiro–Wilk test, and no significant difference was found. Comparison of ERG responses and Arden ratios with maximum standard levels was performed using one-sample Student *t*-test and test of significance was one-tailed. Normal values of standard full-field electroretinograghy in Iranian population with identical method and machine, as reported by Parvaresh et al. were used for the statistical analysis [15]. The normal values of right and left eyes were reported separately; so we analyzed the data according to laterality.

Comparison between clinical examination findings and electrophysiological outcomes were done by the two-tailed independent-sample *t*-test. The level of significance was set at 0.05.

## Results

### Subjects

Forty Iranian male veterans with late complications of SM poisoning were studied. The mean age of the victims was 49.73 ± 8.5 years at the time of study. All the patients had been exposed to SM for one session via gas inhalation. However, the majority did not leave the exposure zone for a couple of hours. The mean duration of exposure was approximately 13.35 ± 8.7 h (1.5–48). The mean percentage of chemical disabilities of the veterans was 45.26 ± 15.4 according to VMAF scale. Reproductive history showed that all the veterans were married men and had children. The mean body mass index (BMI) of patients was 26.28 ± 4.4 kg/m^2^. The group we have selected were exposed in the period of 1987–1988, near 28–29 years after exposure they have included in the study.

### Ocular complications

**Fig. 1:**
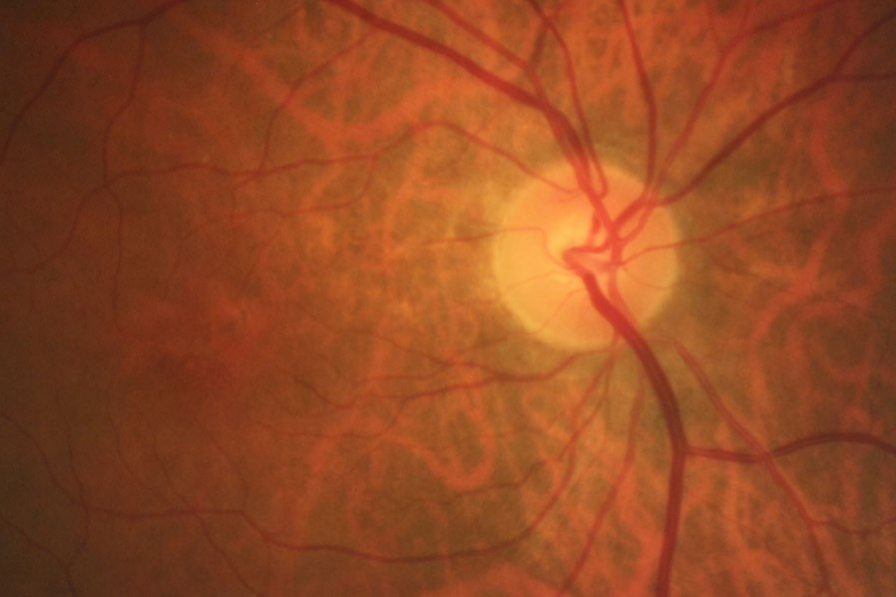
Fundus photograph of the macula of the right eye of one of our patients, showing tessellated fundus and also the pigmentation of macula

Almost all the patients had complaints of eye complications (95%). The most frequent symptoms were as lacrimation (82.5%), foreign body sensation (70%) and dry eye (50%). No patient had a complaint of dark adaptation. Mean visual acuity was 0.098 ± 0.012 LogMAR (logarithm of the Minimum Angle of Resolution). Physical examination revealed bulbar congestion in 22 patients (55%) and corneal sense diminution in 13 patients (32.5%). Slit lamp examination showed iris sponge atrophy of both eyes in 36 patients (90%), diminished tear film in 28 patients (70%), abnormal corneal findings in 19 patients and abnormal limbus in 6 patients (47.5 and 15% respectively). Corneal opacity was observed in 12 patients (30%). The corneal opacity was not significant regarding the severity and position. Furthermore, 3 patients had a history of penetrating keratoplasty for visually significant corneal opacity. No sign of significant lens opacity was present in our study population. Fundus examination showed macular pigmentary changes of both eyes in 37 veterans (92.5%) which was identified as the most important objective finding (Fig 1).

### Electrophysiological findings

There was no statistically significant difference between the two eyes of the patients in terms of all wave amplitudes and implicit times (*p* > 0.05), therefore, 80 eyes of 40 patients were analyzed. The electrophysiological findings of patients and normal values of domestic population are summarized in Table 1.

**Table 1:** Electroretinographic findings of study population compared to domestic normal values

	Right eye						Left eye					
	Amplitude			Implicit time			Amplitude			Implicit time		
	Study findings	Population data	p value (CI 95%)	Study findings	Population data	p value (CI 95%)	Study findings	Population data	p value (CI 95%)	Study findings	Population data	p value (CI 95%)
*Rod*												
b-wave	96.90 ± 78.74	145 ± 54	0.02 (58.949–134.851)	85.11 ± 7.42	92 ± 7	0.0008 (81.534–88.686)	59.40 ± 39.95	142 ± 42	<0.0001 (40.14–78.65)	84.80 ± 6.63	96 ± 8	<0.0001 (81.60–87.99)
*Max response*												
a-wave	120.22 ± 58.84	187 ± 42	p < 0.0001 (101.402–139.038)	20.47 ± 1.67	22 ± 2	<0.0001 (19.936–21.004)	120.22 ± 58.84	192 ± 40	<0.0001 (101.402–139.038)	20.47 ± 1.67	21 ± 2	0.051 (19.936–21.004)
b-wave	270.49 ± 101.90	412 ± 71	p < 0.0001 (237.901–303.079)	43.78 ± 2.48	42 ± 4	0.0001 (42.987–44.573)	245.20 ± 73.12	415 ± 76	<0.0001 (221.815–268.585)	42.80 ± 2.23	43 ± 4	0.573 (42.087–43.513)
*Cone response*												
a-wave	27.28 ± 7.71	30 ± 12	0.0315 (24.814–29.746)	14.26 ± 2.13	15 ± 1	0.0340 (13.579–14.941)	30.24 ± 14.54	31 ± 11	0.7427 (25.59–34.89)	14.45 ± 1.39	15 ± 1	0.0166 (14.005–14.895)
b-wave	70.40 ± 32.48	161 ± 32	<0.0001 (60.012–80.788)	31.52 ± 1.42	31 ± 1	0.0543 (30.99–32.05)	67.31 ± 30.19	162 ± 34	<0.0001 (57.655–76.965)	31.40 ± 1.72	31 ± 1	0.1494 (30.85–31.95)
*30-Hz*												
N1				41.00 ± 11.52	13 ± 1	<0.0001 (37.316–44.684)				41.70 ± 11.06	14 ± 1	<0.0001 (38.163–45.237)
P1				54.43 ± 27.10	26 ± 2	<0.0001 (45.763–63.097)				58.60 ± 11.05	27 ± 2	<0.0001 (55.066–62.134)
N1-P1	54.43 ± 27.10	94 ± 24	p < 0.0001 (45.763–63.097)				58.50 ± 31.61	96 ± 15	<0.0001 (48.391–68.609)			

Rod response ERG findings showed significant decrement in b-wave amplitude (*p* ≤ 0.02) and decrease in implicit time of b-wave (*p* ≤ 0.001) in both eyes when compared to normal population data of the same sex and range of age. The statistical results of these comparisons are summarized in Table 1.

In 30 Hz Flicker response test, significant difference compared to data of normal population in the same range of age and sex was observed in implicit time and amplitude (Table 1).

In EOG recordings, the cut-off point was 1.8 for Arden ration. The mean Arden ratio in the right eyes was 2.13 ± 0.71 and in the left eyes as 2.20 ± 0.52 which showed noninferiority (*p* = 0.0473 and *p* = 0.0035, respectively) compared the normal value of 1.8.

## Discussion

Historically, SM was first synthesized in 1822 by Despretz and modified in 1860 by Guthrie [2]. SM was first used in July 1917 in Ypres, Belgium, during World War I, which eventually led to 1,200,000 SM exposures throughout the war [11]. To date, SM has been inflicted the most casualties among a host of CWAs [1].

SM is a vesicant alkylating agent which can attack and destroy DNA in specific nucleotides and therefore leading to inhibition of DNA, RNA and protein synthesis. Although SM reacts with RNA, proteins and phospholipids, it is mostly known as a DNA alkylating agent which plays an important role in delayed healing [4, 16]. Thus, it can induce further late-onset complications compared to other CWAs and is known as the ‘Capacitating agent’. Evidence also indicates the reactive oxygen species (ROS) and immune reactions against corneal proteins (collagen-mustard compound) that may play a role in SM eye injuries [17, 18].

Eye is one of the most sensitive surface organs in the SM exposure victims. Long-term ocular complications are seen in almost all the survivors. Balali-Mood et al. have previously reported 67.5% ocular surface involvement in 40 Iranian veterans 16–20 years post-exposure [3]. Delayed keratopathy has been mostly discussed recently as a long-term complication [11, 18, 19]. Delayed ulcerative keratopathy may develop in 1% of the exposed patients, leading to late-onset blindness [10, 12]. Lesions are surprisingly recurring even after corneal transplantation [20].

Etezad-Razavi et al. reported long-term ocular complications in 40 SM poisoned veterans 16-20 years after exposure. Itching (42.5%), burning sensation (37.5%), photophobia (30%) and tearing (27.5%) were the most common symptoms. Chronic conjunctivitis and subepithelial opacity were the most abnormal findings in conjunctiva and cornea, respectively [6]. Opacification was observed in lower and central portions of cornea, whereas the upper part was almost protected due to the eyelids [4, 19]. Corneal changes may be more dependent on the dry eye changes induced by the SM exposure effects on the conjunctiva and goblet cells [21, 22]. In our patients mild pigmentary changes of the macula was observed. Beside electrophysiological changes, pigmentary changes mostly in the macula, may another indicator of retinal changes due to mustard gas exposure.

Namazi et al. in an investigation on 3400 files of SM Iranian veterans in VMAF, reported burning sensation (68.65%), photophobia and red eye (63.64%), itching and foreign body sensation (63.43%), dry eye (61.19%), blepharitis (27.61%), and corneal ulcer (11.94%) as common long-term ocular complications [23].

Our study showed a general reduction of retinal photoreceptor function in chronic SM exposure. This effect involves both cone and rod photoreceptors in terms of amplitude and implicit time. These findings in ERG records of SM veterans show that SM intoxication may have late complications on neurologic tissues such as retina. Banin et al. evaluated early-onset effects of nitrogen mustardin an experimental animal retinal model with ERG and showed that retinal function was not affected by the anterior chemical injury [11]. ERG was performed only 6–7 weeks after ocular surface exposure to nitrogen mustard (NM) and ERG was performed mostly to evaluate the toxicity of a scheduled regimen of the consequences of NM exposure. As all the patients had a complete ocular and fundus examination, we are sure that no other pathological finding contributed in this signal reduction. As the retina is not a surface tissue, it may take a longer time for SM to reach the retina via systemic circulation and start its effects leading to retinal functional disturbances.

Our limitations at this study are the small sample, low cooperation for the ocular exams, and lack of similar studies of chemical warfare veterans. As the number of these veterans are small and as they are aging, it seems hard to find cooperative veterans without systemic diseases in which affect the retina. Larger sample size and complying functional with anatomical findings will be required for more accurate interpretation of the findings in future studies. Moreover, the present study compared normative data from other study group. This can be a study limitation. The ideal would be include non SM exposure veterans at same demographic distribution, as controls. Our first study plan was it, however, finding a matched control group from veterans was so difficult that we changed our plan to compare our values, as our routine in ERG reporting, with a normative database by the same facilities and same race and population database.

## Conclusion

In electrophysiological evaluation of forty Iranian sulfur mustard poisoned veterans, delayed toxic effects of SM poisoning were observed in the retina, but not in the retinal pigment epithelium layer. As the retina is a neural tissue, long-term effects of SM on neural tissues are presumed. To our knowledge, this is the first report on the delayed-onset functional retinal changes in patients with exposure to SM. Other reports were mostly on ocular surface findings, either early or late.
